# Geniposide Possesses the Protective Effect on Myocardial Injury by Inhibiting Oxidative Stress and Ferroptosis *via* Activation of the Grsf1/GPx4 Axis

**DOI:** 10.3389/fphar.2022.879870

**Published:** 2022-05-05

**Authors:** Yuehong Shen, Xindong Wang, Xinyu Shen, Yue Wang, Shulin Wang, Yunyun Zhang, Xiaoming Yao, Yijiao Xu, Ming Sang, Jiamin Pan, Yu Qin, Qian Zhou, Jianping Shen

**Affiliations:** ^1^ Affiliated Hospital of Integrated Traditional Chinese and Western Medicine, Nanjing University of Chinese Medicine, Nangjing, China; ^2^ Department of Biostatistics, School of Global Public Health, New York University, New York, NY, United States; ^3^ Zhenjiang Hospital Affiliated to Nanjing University of Chinese Medicine (Zhenjiang Hospital of Traditional Chinese Medicine), Zhenjiang, China

**Keywords:** geniposide, myocardial ischemic injury, oxidative stress, ferroptosis, iron accumulation, lipid peroxidation

## Abstract

Reactive oxygen species (ROS) produced in the ischemic myocardium can induce cardiomyocyte injury and death, resulting in cardiac remodeling. Ferroptosis, known as a newly type of cell death caused by iron-dependent oxidative stress, which is an essential death mechanism in cardiomyocytes. However, it is unclear whether oxidative stress products can further induce ferroptosis and aggravate cardiomyocyte injury. Geniposide (GEN), a major active component of *Gardenia jasminoides J. Ellis*, possesses the natural antioxidant activity and cardioprotective effect. Herein, we evaluated the role of ferroptosis in myocardial oxidative injury and the protective effect of GEN on myocardial ferroptosis. We first detected iron overload, massive ROS, and lipid peroxidation in ferric ammonium citrate (FAC)-treated cardiomyocytes, which were typical characteristics of ferroptosis. The iron overload-induced oxidative stress and ferroptosis aggravated cardiomyocyte injury, which were significantly alleviated by GEN treatment. Similar phenotypic changes of ferroptosis were consistently discovered in hydrogen peroxide (H_2_O_2_)-induced cells, which were reversed by GEN treatment as well. Interestingly, the RNA-binding protein Grsf1, which directly upregulated Gpx4 at the translational level, was activated by GEN following myocardial oxidative injury. The specific knockdown of Grsf1 increased their sensitivity to ferroptosis and weakened the cardioprotective effect of GEN in H_2_O_2_-treated cardiomyocytes. Moreover, GEN treatment reduced iron overload and lipid peroxidation in myocardial infarction (MI) rats, thereby fighting against the cardiac ischemic injury. Collectively, our study revealed the pathogenesis of oxidative stress and ferroptosis associated with myocardial ischemia, and indicated the antioxidant and anti-ferroptosis effects of GEN on preventing myocardial injury by activating the Grsf1/GPx4 axis, serving as a potential therapeutic target.

## Introduction

Ischemic heart disease is the major cardiovascular event, which has been globally concerned for its high morbidity, mortality, and disability rates (Meng Khoo and Tai, 2014; Brown et al., 2015). Studies have shown that oxidative stress is involved in the development of myocardial ischemia, leading to myocardial fibrosis, left ventricular dysfunction, and hypertrophy (Ferrari et al., 2004). Reactive oxygen species (ROS) produced in the ischemic area are favorable to a microenvironment of oxidative stress, in which the damaged myocardium and cell membrane eventually cause cardiac dysfunction and heart failure (Misra et al., 2009). Thus, targeting oxidative stress is an effective way to prevent ischemic heart disease.

Oxidative stress mediates myocardial injury by inducing different types of cardiomyocyte death, which is mainly through cell apoptosis and necrosis. Apoptosis is a caspase-3-dependent form of programmed cell death that mainly manifests as cell shrinkage, chromatin condensation, nuclear fragmentation, and apoptotic body formation (Zhang et al., 2019). Necrosis has long been considered as an accidental, uncontrolled form of cell death, which is characterized by the expansion of organelles, plasma membrane rupture, and release of the cell contents (Nunes et al., 2014). However, recent studies have demonstrated that necrosis can be tightly regulated by various signaling pathways, serving as regulated necrosis (Martin-Sanchez et al., 2018). Among the various types of regulated necrosis, ferroptosis, a newly discovered non-apoptotic cell death, is caused by iron overload and lipid peroxidation accumulation, leading to oxidative damage (Dixon et al., 2012; Zhu et al., 2020). Free iron catalyzes the overproduced ROS *via* the Fenton and Haber-Weiss reactions, resulting in cell injury (Valko et al., 2016). In the early 1980s, Sullivan (1981) for the first time postulated the iron-heart hypothesis, revealing the importance of iron stores in the human body. Since the discovery of ferroptosis, great efforts have been made on exploring the pathogenesis of ferroptosis in heart disease. Fang et al. (2019) demonstrated that ferroptosis underlies doxorubicin-induced cardiac toxicity and ischemia/reperfusion-induced cardiomyopathy, revealing the vital role of ferroptosis in the progression of heart disease. Notably, the content of low-molecular-weight iron in cardiomyocytes significantly increases during myocardial ischemia (Voogd et al., 1992). Abnormally increased myocardial iron causes lipid peroxidation, further triggering massive production of ROS and oxidative injury, which may be a critical factor in myocardial remodeling (Kobayashi et al., 2018).

Myocardial ferroptosis is mainly regulated by iron metabolism and antioxidant signaling pathways. As a bridge linking oxidative stress and ferroptosis, ROS play a central role in the execution of cell death (Park and Chung, 2019). Loss of glutathione peroxidase 4 (GPx4) activity is one of the key factors resulting in ferroptosis. GPx4 is an essential regulator of glutathione (GSH) metabolism that specifically scavenges phospholipid hydrogenperoxide, thereby exerting anti-ferroptosis and antioxidant effects (Feng et al., 2020b; Ursini and Maiorino, 2020). Park et al. (2019) demonstrated that the downregulation of GPx4 during myocardial infarction (MI) aggravates ferroptosis injury in myocardium. Guanine-rich RNA sequence binding factor 1 (Grsf1) is a member of the RNA-binding protein family that regulates RNA metabolism (Ufer, 2012). It is reported that Grsf1-mediated translational regulation of GPx4 expression is essential for embryonic brain development (Ufer et al., 2008). Grsf1 upregulates the mRNA level of mitochondrial GPx4 through directly targeting it, leading to a decrease in the ROS levels. We speculated that Grsf1 may inhibit myocardial ferroptosis through its antioxidant effects by upregulating GPx4 expression.

Dried mature fruit of *Gardenia jasminoides J. Ellis* (termed Zhizi in Chinese) is widely used in the treatment of cardiovascular diseases. Its pharmacological actions include the regulation of atherosclerosis, protection of cardiomyocytes, suppression of myocardial fibrosis, and decreasing blood pressure and blood lipid levels (Bu et al., 2020; E et al., 2021). Geniposide (GEN) is a major active component extracted from *Gardenia jasminoides J. Ellis*, which possesses the natural antioxidant activity and cardioprotective effect (Luo et al., 2020). Numerous studies have demonstrated that GEN protects against myocardial ischemia by suppressing the oxidative stress response (Lu et al., 2018; Zhou et al., 2020). Wu (2012) demonstrated that GEN can scavenge hydroxyl free radicals and inhibit lipid peroxidation in heart tissue homogenate *in vitro*. However, to our knowledge, a protective effect of GEN on myocardial ferroptosis has not been reported yet. It is of significance to explore the cardioprotective role of GEN through inhibiting ferroptosis-related oxidative stress pathways.

In this study, we first investigated the pathogenesis and effect of ferroptosis induced by iron overload on cardiomyocytes and the potential involvement of iron overload-induced ferroptosis in the cardioprotective effect of GEN. Next, we established an *in vitro* oxidative stress model to simulate myocardial oxidative injury and examined ferroptosis-related changes in cardiomyocytes, and explored whether H_2_O_2_-induced ferroptosis was involved in the effect of GEN-mediated cardioprotection. Using ferroptosis inhibitors ferrostatin-1 (Fer-1) and deferoxamine (DFO) as positive controls, expression levels of ferroptosis-related proteins were measured. After knockdown of Grsf1 in H_2_O_2_-treated cardiomyocytes, ferroptosis markers were detected to validate the role of Grsf1/GPx4 axis in the protective effect of GEN against ferroptosis and oxidative stress. We further constructed an *in vivo* MI rat model and explored the interventional effect of GEN against myocardial ferroptosis by measuring ferroptosis markers and pathological changes of myocardial ischemia.

## Materials and Methods

### Cell Lines and Culture Conditions

Primary cardiomyocytes were collected from the neonatal Institute of Cancer Research (ICR) mice with 1–3 days old, as previously reported (Chen et al., 2018). In brief, neonatal mice were sacrificed by cervical dislocation and their hearts including the ventricles were aseptically collected by chest operations, and stored in pre-cold Ca^2+^/Mg^2+^-free Hank’s balanced salt solution. The ventricles were cut into small pieces and subjected to 0.05% trypsin digestion, with serial cycles of agitation. The supernatant was collected and fetal bovine serum (FBS) was added to a final concentration of 10%. The mixture was centrifuged at 100 × g for 10 min, and the pelleted cells were resuspended in DMEM/F12 supplemented with 10% FBS, 100 U/ml penicillin, and 100 μg/ml streptomycin. The cells were incubated in a humid incubator (37°C, 5% CO_2_) for 2 h to obtain cardiomyocytes and nonmyocytes. To prevent the proliferation of nonmyocytes, 100 μmol/L bromodeoxyuridine was added during the first 48 h of cardiomyocyte culture.

Rat cardiac H9c2 cells were purchased from the American Type Culture Collection (Manassas, VA). They were cultured in DMEM supplemented with 10% FBS, 4.5 mg/ml high glucose, 100 U/ml penicillin, 100 μg/ml streptomycin, and 2 mM L-glutamine in a humid incubator with 5% CO_2_ at 37°C.

### Cell Counting Kit-8 (CCK-8) Assay and Lactate Dehydrogenase (LDH) Assay

CCK-8 assay (MR1003, Sciben, Nanjing, China) was used per the manufacturer’s instructions to measure cell viability. Primary cardiomyocytes and H9c2 cells were seeded in a 96-well culture plate (1 × 10^4^ cells/well) and incubated in a humid incubator with 5% CO_2_ at 37°C. Cells were exposed to different concentrations of FAC or H_2_O_2_ for 24 h and either treated with various doses of GEN for 24 h or not. Then, 10 μl of CCK-8 solution was added to each well and the plates were incubated for another 2 h. The absorbance at 450 nm was measured using a Victor X3 Light Plate Reader (PerkinElmer, Waltham, MA).

LDH assay was performed using the commercial kit (A020-2, Jiancheng, Nanjing, China) to assess cytotoxicity. Cells were cultured in medium containing 10% FBS, and 10 µl of culture was used for the LDH assay. LDH reaction mix was added per well and cells were incubated at room temperature for 30 min. LDH activity was quantified by measuring the optical density at 450 nm using the Victor X3 Light Plate Reader.

### Cell Transfection

H9c2 cells were seeded in 96-well culture plates (1 × 10^4^ cells/well) in antibiotic-free DMEM supplemented with 10% FBS for 24 h. When the cells reached 70–90% confluence, they were transfected with a shRNA targeting Grsf1 (shGrsf1) or a control shRNA harboring the green fluorescent protein (shGFP) sequence, using Lipofectamine 2000 reagent (Invitrogen, United States) per the manufacturer’s instructions. The forward specific sequence of shGrsf1 was 5′-AAT​TCC​CGC​GAT​GCC​TTG​ATT​GAA​ATG​GTG​CAA​GAG​ACC​ATT​TCA​ATC​AAG​GCA​TCG​CTT​TTT​G-3′ and the downward sequence was 5′-GAT​CCA​AAA​AGC​GAT​GCC​TTG​ATT​GAA​ATG​GTC​TCT​TGC​ACC​ATT​TCA​ATC​AAG​GCA​TCG​CGG​G-3′.

### ROS Assay

Intracellular ROS levels were detected using a 2′,7′-dichlorofluorescein diacetate (DCFH-DA) probe (MR1008, Sciben, Nanjing, China). Cells were seeded in a 96-well plate (1 × 10^4^ cells/well) and incubated with a DCFH-DA solution at a final concentration of 10 μm at 37°C for 20 min, followed by three times of washing with serum-free cell culture medium. The fluorescence intensity was imaged using a fluorescence microscope with excitation at 485 nm and emission at 535 nm.

### Cell Malondialdehyde (MDA), Superoxide Dismutase (SOD), and Superoxide Anion (SA) Assay

MDA (A003-4, Jiancheng, Nanjing, China), SOD (A001-1, Jiancheng, Nanjing, China), and SA (BC1295, Solarbio, Beijing, China) levels were assayed using commercial kits per manufacturers’ instructions.

### Reactivity of Phen Green SK (PGSK) With Ferrous Ions (Fe^2+^)

Fe^2+^ were reactivated and quantified using the fluorescent heavy metal indicator, PGSK (25393, Cayman, Ann Arbor, United States). PGSK fluorescence was quenched upon interaction with Fe^2+^. In brief, cells were loaded with PGSK (20 μmol/L) dissolved in DMSO for 10 min. Then, PGSK was removed by washing twice. The fluorescence intensity was imaged using a fluorescence microscopy with excitation at 507 nm and emission at 532 nm.

### Analysis of the Mitochondrial Membrane Potential (ΔΨm) Using JC-1

JC-1 (MR1009, Sciben, Nanjing, China) is a fluorescent probe that can sensitively reflect changes in ΔΨm. Treated primary cardiomyocytes and H9c2 cells were incubated with 0.5 ml of JC-1 staining solution at 37°C for 20 min. Then, the cells were washed with JC-1 staining buffer and captured using a fluorescence microscope.

### Animals

SPF-level adult male Sprague-Dawley (SD) rats weighing 220–250 g were purchased from Experimental Animal Business Department of Shanghai Institute of Planned Parenthood Research (Shanghai, China). The rats were housed in standard cages in a temperature- and humidity-controlled room (22–24°C; relative humidity 40–60%) under 12-h light/dark cycles and they were given free access to food and water. All animal experiments were approved by the Ethics Committee of Jiangsu Province Academy of Traditional Chinese Medicine (No. AEWC-20181205-65), and they were conducted following the National Institutes of Health’s Guide for the Care and Use of Laboratory Animals.

### MI Rat Model Establishment and Experimental Groups

Male SD rats (8–10 weeks) were randomly divided into Sham, MI, MI + DFO, and MI + GEN groups. After a 12-h fasting, all animals were anesthetized with 10% chloral hydrate (4 ml/kg, intraperitoneally). The rats were placed in a supine position on a fixed plate and intubated and ventilated with a small-animal ventilator (ALC-V8S, Shanghai Alcott Biotech Co., Ltd., Shanghai, China). After exposing the chest cavity around the third intercostal space, the left anterior descending (LAD) coronary artery between the pulmonary conus and the left atrial appendage was ligated using a sterile 7–0 silk suture. Ligation was deemed successful when the left ventricle wall turned pale and the electrocardiogram (ECG) showed an elevated ST segment. Animals in the Sham group underwent the same operation without ligation of the LAD coronary artery. Finally, 32/42 (76.19%) rats in the four groups survived and they were used in the animal experiments (*n* = 8/group). The rats were monitored for 24 h after surgery. Those in the MI + DFO and MI + GEN groups received intraperitoneal injections of DFO (Sigma-Aldrich, United States; 100 mg/kg, dissolved in normal saline) or GEN (Shanghai Winherb Medical Science Co., Ltd., China; 50 mg/kg, dissolved in normal saline) once a day for 7 days. Heart tissues and blood were harvested after euthanasia and preserved for biochemical analysis and examination.

### 2,3,5-Triphenyltetrazolium Chloride (TTC) Staining for Infarct Size Measurement

After humane sacrifice, rat hearts were collected and frozen at −80°C for 15 min. Then the hearts were cut into 1–2-mm sections, which were prepared from the same heart area in all groups for comparison of the infarct size, and stained with TTC (Solarbio, Beijing, China) solution in a constant temperature box at 37°C for 30 min. Infarcted myocardium was not or faintly stained, whereas normal tissues were stained red. After staining, the sections were washed with distilled water, fixed in 4% paraformaldehyde, and captured and analyzed using ImageJ. The results were expressed as the percentage of the infarcted area in the total area of the section.

### Measurement of Serum Creatine Kinase-MB (CK-MB), and Lactate Dehydrogenase (LDH)

The serum CK-MB and LDH levels were measured by an automatic biochemical analyzer (C8000 Roche, Hoffmann-La Roche Inc., Switzerland).

### Hematoxylin and Eosin (H&E) Staining

Rat left ventricles, including lesions and normal areas below the ligation site, were fixed in paraformaldehyde for 48 h, embedded in paraffin, and sectioned. The sections were incubated in hematoxylin staining solution for 5 min, followed by washing. Then, the sections were stained in eosin solution for 5 min. After dehydration and mounting, the sections were observed and captured using a microscope to assess histopathological changes.

### Prussian Blue Staining

Prussian blue staining was performed to detect iron deposition in myocardial tissues. Heart sections were incubated in the mixture containing the isodose Prussian blue dye A and Prussian blue dye B for 1 h, followed by washing twice in ddH_2_O. Prussian blue dye C was used to stain the nuclei. After dehydration and sealing, the sections were captured under a microscope.

### Measurement of Tissue MDA and GSH

MDA content and GSH level in heart tissues were determined using MDA Assay Kit (A003-1, Jiancheng, Nanjing, China) and GSH Assay Kit (A006-2, Jiancheng, Nanjing, China) in accordance with the manufacturer’s instructions, respectively.

### Western Blotting Analysis

Cardiac cells and tissue homogenates were lysed with Western blotting analysis buffer (Beyotime Biotechnology, Shanghai, China) to extract total proteins. Protein concentrations were determined using a Nanodrop 1000 instrument (Thermo Scientific, United States), and they were adjusted to the same concentration. The samples were boiled with 5 × loading buffer (Beyotime Biotechnology, Shanghai, China) and subjected to 10% SDS-PAGE. The proteins were transferred to polyvinylidene fluoride membranes (PVDF; Merk Millipore Ltd., Darmstadt, Germany) at 300 mA. The membranes were blocked with 5% non-fat milk in TBST at room temperature for 1 h, followed by incubation with primary antibodies against prostaglandin-endoperoxide synthase 2 (Ptgs2; sc-166475, Santa Cruz Biotechnology; ab15191, Abcam), Grsf1 (ab205531, Abcam), GPx4 (ab125066, Abcam), transferrin receptor 1 (Tfr1; sc-59112, Santa Cruz Biotechnology), ferritin heavy chain (Fth1; Santa Cruz Biotechnology), and Gapdh (AT15705, Sciben) at 4°C overnight. On the following day, the membranes were washed with TBST three times and incubated with the corresponding secondary antibodies at room temperature for 2 h. Protein bands were visualized using ultrahigh sensitivity ECL chemiluminescence reagent (Beyotime Biotechnology, Shanghai, China) and an automated chemiluminescence image analysis system (Tanon 5200, Shanghai, China). Band intensities were quantified using ImageJ.

### Statistical Analysis

All data were expressed as mean ± standard deviation. Graphpad Prism 8.0 software (San Diego, CA, United States) and Microsoft Excel were used for statistical analysis. Differences between groups were compared by the Student’s *t*-test, and those among three or more groups were compared using one-way ANOVA. A *p-*value less than 0.05 was considered as statistically significant.

## Results

### GEN Suppresses Iron Overload-Induced Ferroptosis and Oxidative Stress Injury in Cardiomyocytes

**FIGURE 1:**
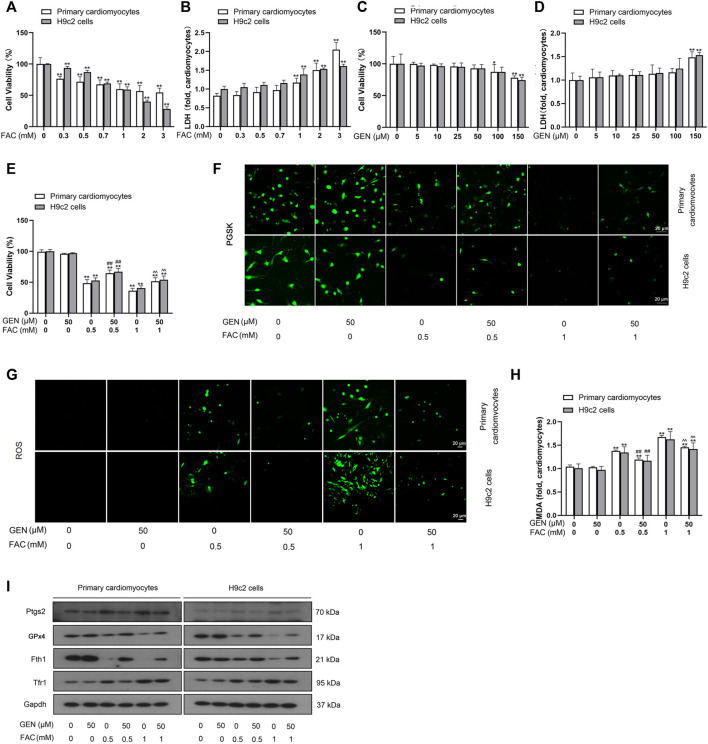
FAC induces ferroptosis and oxidative stress injury in cardiomyocytes, which are reversed by GEN treatment. **(A,B)** Viability and toxicity of primary cardiomyocytes and H9c2 cells treated with different concentrations of FAC for 24 h. **(C,D)** Viability and toxicity of primary cardiomyocytes and H9c2 cells treated with various doses of GEN. **(E)** Viability of cardiomyocytes treated with 50 μm GEN after treatment with 0.5 or 1 mM FAC. **(F)** Iron level in FAC-induced cells with GEN treatment. **(G)** ROS generation (green) in FAC-pretreated cells treated with GEN. **(H)** MDA level in FAC-treated cells after treatment with GEN. **(I)** Protein expression levels of Ptgs2, GPx4, Fth1, and Tfr1 in cardiomyocytes pretreated with FAC and then treated with GEN. Data were expressed as mean ± SD (*n* = 5). *p < 0.05, **p < 0.01 vs. Control group; ^##^ *p* < 0.01 vs. 0.5 mM FAC group; ^ ^*p* < 0.01 vs. 1 mM FAC group. Scale bar = 20 µm.

To validate whether iron overload is essential for triggering myocardial ferroptosis and how it affects myocardial cells, FAC was used to induce iron overload in cardiomyocytes. Both primary cardiomyocytes and H9c2 cells were exposed to 0–3 mM FAC for 24 h, followed by assessment of cell viability and toxicity by the CCK-8 assay and LDH assay, respectively. The CCK-8 result showed that FAC treatment decreased the cell viability in a dose-dependent manner (Figure 1A). As shown in Figure 1B, the release of LDH in cells with FAC induction dose-dependently increased, suggesting the increases of cytotoxicity. Similarly, we assessed viability and toxicity in primary cardiomyocytes and H9c2 cells induced with 0–150 μM GEN (Figures 1C,D). As shown in Figure 1E, treatment with 50 μM GEN restored the cell viability reduction induced by 0.5 mM or 1 mM FAC. Based on these results, we selected two concentrations of FAC at 0.5 and 1 mM and one dose of GEN at 50 μM for further experiments.

Iron accumulation was firstly detected using PGSK diacetate, which reacts with various metal ions. Intracellular PGSK fluorescence is readily quenched upon reaction with iron ions. Compared with control cells, FAC induction dose-dependently quenched the PGSK fluorescence in cardiomyocytes, which was reversed by 50 μM GEN treatment (Figure 1F, Supplementary Figure S1). Given that lipid peroxidation is a characteristic marker of ferroptosis, we measured the production of ROS and MDA content in FAC-induced myocardial cells. Compared with control cells, cells cultured with FAC showed a higher fluorescence intensity in a concentration-dependent manner, indicating higher ROS levels (Figure 1G, Supplementary Figure S1). However, GEN treatment significantly suppressed the overproduction of ROS in FAC-induced cells (Figure 1G, Supplementary Figure S1). Similar result was observed in the assessment of MDA content (Figure 1H). We next measured the protein expressions of ferroptosis markers, including Ptgs2, GPx4, Fth1, and Tfr1. Fth1 and GPx4 were downregulated, while Ptgs2 and Tfr1 were upregulated in cells treated with 0.5 or 1 mM FAC when compared with control cells (Figure 1I, Supplementary Figure S1). However, GEN treatment reversed the expression trends of the above proteins in FAC-treated cardiomyocytes (Figure 1I, Supplementary Figure S1). Collectively, these findings suggested that iron overload caused oxidative damage to cardiomyocytes by triggering ferroptosis, and GEN presented an inhibitory effect on iron overload-mediated lipid peroxidation and ferroptosis.

### GEN Alleviates H_2_O_2_-Induced Ferroptosis Injury in Cardiomyocytes

**FIGURE 2:**
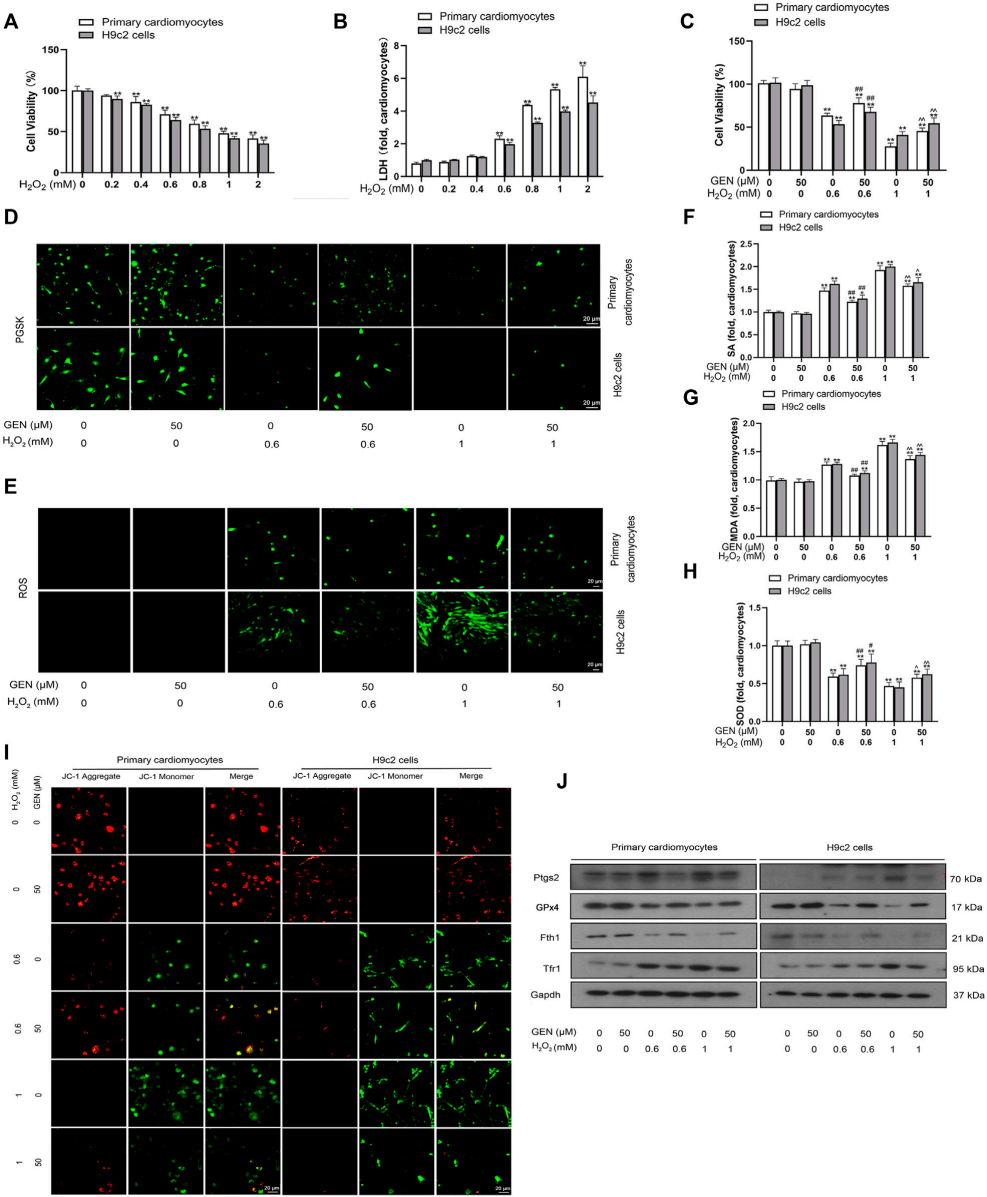
GEN rescues H_2_O_2_-induced ferroptosis injury in cardiomyocytes. **(A,B)** Viability and toxicity of primary cardiomyocytes and H9c2 cells treated by different concentrations of H_2_O_2_ for 24 h. **(C)** Viability of cardiomyocytes treated with 50 μm GEN after pretreatment with 0.6 or 1 mM H_2_O_2_. **(D)** Effect of GEN on iron accumulation in H_2_O_2_-induced cells. **(E–H)** Effects of GEN on lipid ROS, SA, MDA, and SOD levels in H_2_O_2_-induced cells. **(I)** JC-1 fluorescence in H_2_O_2_-induced cardiomyocytes treated with GEN. **(J)** Protein expression levels of Ptgs2, GPx4, Fth1, and Tfr1 in H_2_O_2_-induced cells treated with GEN. Data were expressed as mean ± SD (*n* = 5). *p < 0.05, **p < 0.01 vs. Control group; ^#^ *p* < 0.05, ^##^ *p* < 0.01 vs. 0.6 mM H_2_O_2_ group; ^*p* < 0.05, ^  ^*p* < 0.01 vs. 1 mM H_2_O_2_ group. Scale bar = 20 µm.

When myocardial ischemia occurs, the hypoxic myocardium is in an oxidative microenvironment. To investigate the effect of oxidative stress on ferroptosis, we simulated the oxidative microenvironment of cardiomyocytes by H_2_O_2_ induction and observed the effect of GEN on injured cardiomyocytes. CCK-8 and LDH assay were performed to measure cell viability and toxicity, respectively. Induction of 0–2 mM H_2_O_2_ for 24 h dose-dependently lowered cell viability in cardiomyocytes (Figure 2A). Besides, H_2_O_2_ treatment increased the LDH level and induced cytotoxicity in a concentration-dependent manner (Figure 2B). Compared with those of controls, 0.6 mM H_2_O_2_ induction lowered cell viability to 29.14 and 35.93% in primary cardiomyocytes and H9c2 cells, respectivaely, which was reduced by 52.10 and 58.28%, respectively at 1 mM H_2_O_2_. The effects of 0.6 and 1 mM H_2_O_2_ on cell viability were rescued by treatment with 50 μM GEN (Figure 2C).

As a prerequisite for ferroptosis, iron accumulation was detected using PGSK diacetate. PGSK fluorescence was quenched in 0.6 or 1 mM H_2_O_2_-treated primary cardiomyocytes and H9c2 cells compared that of with control cells (Figure 2D, Supplementary Figure S2). However, treatment with 50 μM GEN restored the fluorescence intensity in cells induced with 0.6 or 1 mM H_2_O_2_ (Figure 2D, Supplementary Figure S2). ROS, SA, and MDA are all products of oxidative stress and lipid peroxidation that contribute to the progression of ferroptosis to a certain extent. We found that the fluorescence intensity of ROS increased in H_2_O_2_-treated cardiomyocytes in a concentration-dependent mannner compared to that of control cells, which was strongly suppressed by GEN treatment (Figure 2E, Supplementary Figure S2). Similarly, pretreatment with H_2_O_2_ dose-dependently increased SA and MDA levels, whereas GEN significantly suppressed the upregulation of SA and MDA production (Figures 2F,G). Importantly, SOD activity in H_2_O_2_-treated cells significantly increased by GEN intervention, suggesting that GEN regulated ferroptosis in cardiomyocytes by activating the antioxidant system (Figure 2H). Mitochondrial dysfunction is an early feature of ferroptosis that can be evaluated by measuring the mitochondrial membrane potential (ΔΨm). JC-1 is a fluorescent probe with red fluorescence signal in normal cells. The loss of ΔΨm triggers the shift of JC-1 fluorescence signal from red to green (JC-1 monomers). We found that JC-1 monomers increased in H_2_O_2_-treated cells compared with control cells, indicating a decrease in mitochondrial ΔΨm (Figure 2I). As expected, GEN reduced this effect (Figure 2I). To unravel the effect of GEN on H_2_O_2_-induced myocardial ferroptosis further, protein expressions of ferroptosis markers were measured. Compared with those of control cells, Ptgs2 and Tfr1 were upregulated, whereas Fth1 and GPx4 expression were downregulated in H_2_O_2_-induced cells, and GEN reverted these expression trends (Figure 2J, Supplementary Figure S2). Fer-1 and DFO are currently known as ferroptosis antagonists. H_2_O_2_-induced cardiomyocytes were treated with Fer-1 or DFO, and protein expression changes of Ptgs2, GPx4, Fth1, and Tfr1 were reversed either by the treatment of Fer-1 or DFO alone or in combination with GEN, suggesting that ferroptosis can be a new target for protecting against myocardial injury (Supplementary Figures S3, S4).

### GEN Attenuates H_2_O_2_-Induced Myocardial Ferroptosis Through the Grsf1/GPx4 Pathway

**FIGURE 3:**
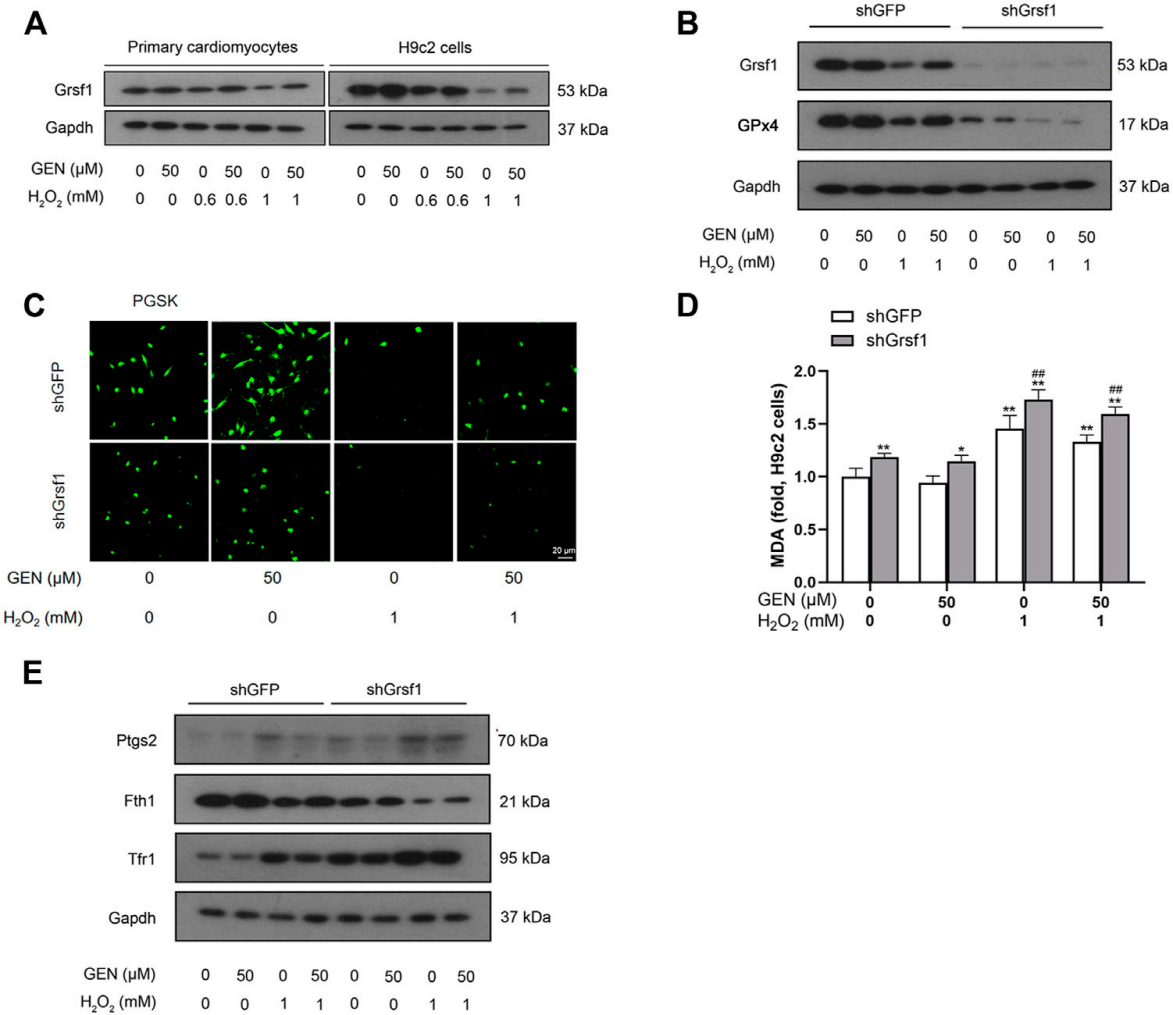
Knockdown of Grsf1 sensitizes cardiomyocytes to H_2_O_2_-induced ferroptosis and weakens the protective effect of GEN against myocardial injury. **(A)** Protein expression of Grsf1 in H_2_O_2_-induced primary cardiomyocytes and H9c2 cells treated with GEN. **(B)** Effect of GEN on protein expressions of Grsf1 and GPx4 in H_2_O_2_-induced H9c2 cells after knockdown of Grsf1. **(C,D)** Iron level and MDA content in H_2_O_2_-induced H9c2 cells after knockdown of Grsf1 and treatment of GEN. **(E)** Effect of GEN on the expressions of the ferroptosis-related proteins Ptgs2, Fth1, and Tfr1 in H_2_O_2_-induced H9c2 cells after knockdown of Grsf1. Data were expressed as mean ± SD (*n* = 5). *p < 0.05, **p < 0.01 vs. shGFP control group; ^#^ *p* < 0.05, ^##^ *p* < 0.01, shGrsf1 group vs. shGFP group. Scale bar = 20 µm.

Grsf1, a member of the RNA-binding protein family, plays an important role in the regulation of GPx4 expression. We found that Grsf1 expression was downregulated in H_2_O_2_-induced myocardial ferroptosis, an effect that was rescued by GEN treatment (Figure 3A, Supplementary Figure S5). To investigate whether the effect of GEN in regulating myocardial ferroptosis requires the activation of Grsf1, shRNA was used to silence Grsf1 in H_2_O_2_-treated H9c2 cells. Western blotting revealed that Grsf1 was significantly downregulated in H_2_O_2_-induced H9c2 cells transfected with shGrsf1 compared with that of negative controls (Figure 3B, Supplementary Figure S5). Surprisingly, GPx4 expression was also significantly suppressed in H_2_O_2_-induced cells with Grsf1 knockdown, suggesting that Grsf1 positively regulated GPx4. GEN treatment did not significantly upregulate Grsf1 and GPx4 in H_2_O_2_-induced cells with Grsf1 knockdown (Figure 3B, Supplementary Figure S5).

Next, we measured several biomarkers of ferroptosis. Intracellular Fe^2+^ levels were measured using PGSK diacetate. As shown in Figure 3C, H_2_O_2_-induced cells with Grsf1 knockdown showed a rapid decrease in fluorescence, suggesting the stimulated release of Fe^2+^. Interestingly, GEN treatment failed to significantly suppress iron accumulation as indicated by a slightly increased fluorescence intensity (Figure 3C, Supplementary Figure S6). Given the important role of lipid peroxidation in myocardial ferroptosis, we measured the production of MDA. As expected, MDA level significantly increased in H_2_O_2_-induced H9c2 cells with Grsf1 knockdown, and this response was mildly suppressed by GEN treatment (Figure 3D). Subsequently, we detected the expression of Ptgs2, which was significantly upregulated in response to H_2_O_2_ treatment in cells with Grsf1 knockdown (Figure 3E, Supplementary Figure S6). We also measured expression levels of several iron-related proteins, including Tfr1 and Fth1. Significantly downregulated Fth1 and upregulated Tfr1 were observed in H_2_O_2_-induced cells with Grsf1 knockdown compared with those transfected with shGFP (Figure 3E, Supplementary Figure S6). However, GEN treatment did not significantly reverse the expressions of Ptgs2, Fth1, and Tfr1 in H_2_O_2_-induced cells with Grsf1 knockdown (Figure 3F, Supplementary Figure S6). Taken together, these findings indicated that silence of Grsf1 renders cardiomyocytes susceptible to H_2_O_2_-mediated ferroptosis, and the protective effects of GEN on injuried myocardium were diminished upon Grsf1 knockdown, indicating that GEN may inhibit oxidative stress and ferroptosis injury *via* activating the Grsf1/GPx4 pathway.

### GEN Blocks Myocardial Ferroptosis in MI Rats

**FIGURE 4:**
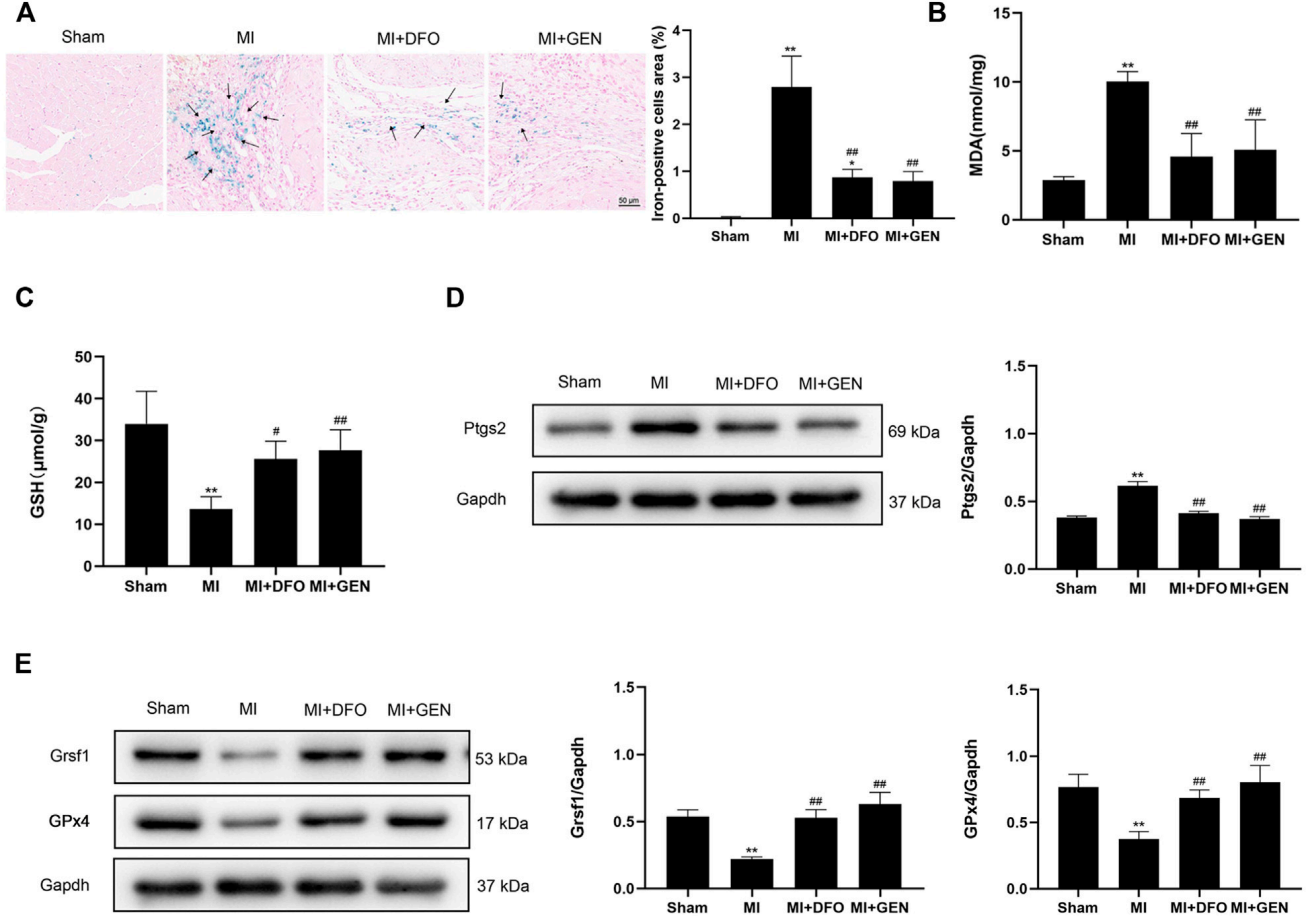
GEN inhibits MI-mediated ferroptosis. **(A)** Prussian blue staining of iron deposition in rat heart tissues. **(B,C)** MDA and GSH contents in rats of Sham, MI, MI + DFO, and MI + GEN group. **(D)** Expression levels of the ferroptosis marker Ptgs2 in rat heart tissues. **(E)** Grsf1 and GPx4 expression levels in the heart tissues. Data were expressed as mean ± SD (*n* = 8). *p < 0.05, **p < 0.01 vs. Sham group; ^#^ *p* < 0.05, ^##^ *p* < 0.01 vs. MI group. Scale bar = 50 µm.

MI can lead to myocardial injury and the subsequent heart failure. ROS release in the ischemic area results in abnormal iron metabolism, which may be associated with myocardial ferroptosis (Ravingerova et al., 2020). To confirm the role of ferroptosis in MI and the protective effect of GEN on the ischemic myocardium, we established an acute MI model in rats through LAD ligation. Prussian blue staining revealed a strong iron deposition in MI rats compared with Sham rats (Figure 4A). Of note, treatment with DFO or GEN reduced the number of iron-positive cells compared with that in the MI group (Figure 4A). We next measured the levels of lipid peroxidation product MDA and the antioxidant GSH in rats. Higher MDA content and lower GSH level were observed in MI rats than those of Sham rats. GEN or DFO intervention significantly reversed these changes, suggesting the suppressed oxidative stress (Figures 4B,C). Next, we measured the expression level of Ptgs2, which was significantly higher in MI rats than that of Sham rats (Figure 4D). Lower expression levels of Grsf1 and GPx4 were detected in MI rats than those of Sham rats (Figure 4E), which were significantly suppressed by the treatment of GEN or DFO (Figures 4D,E).

### Inhibition of Ferroptosis With GEN Attenuates the Cardiac Pathological Damage in MI Rats

**FIGURE 5:**
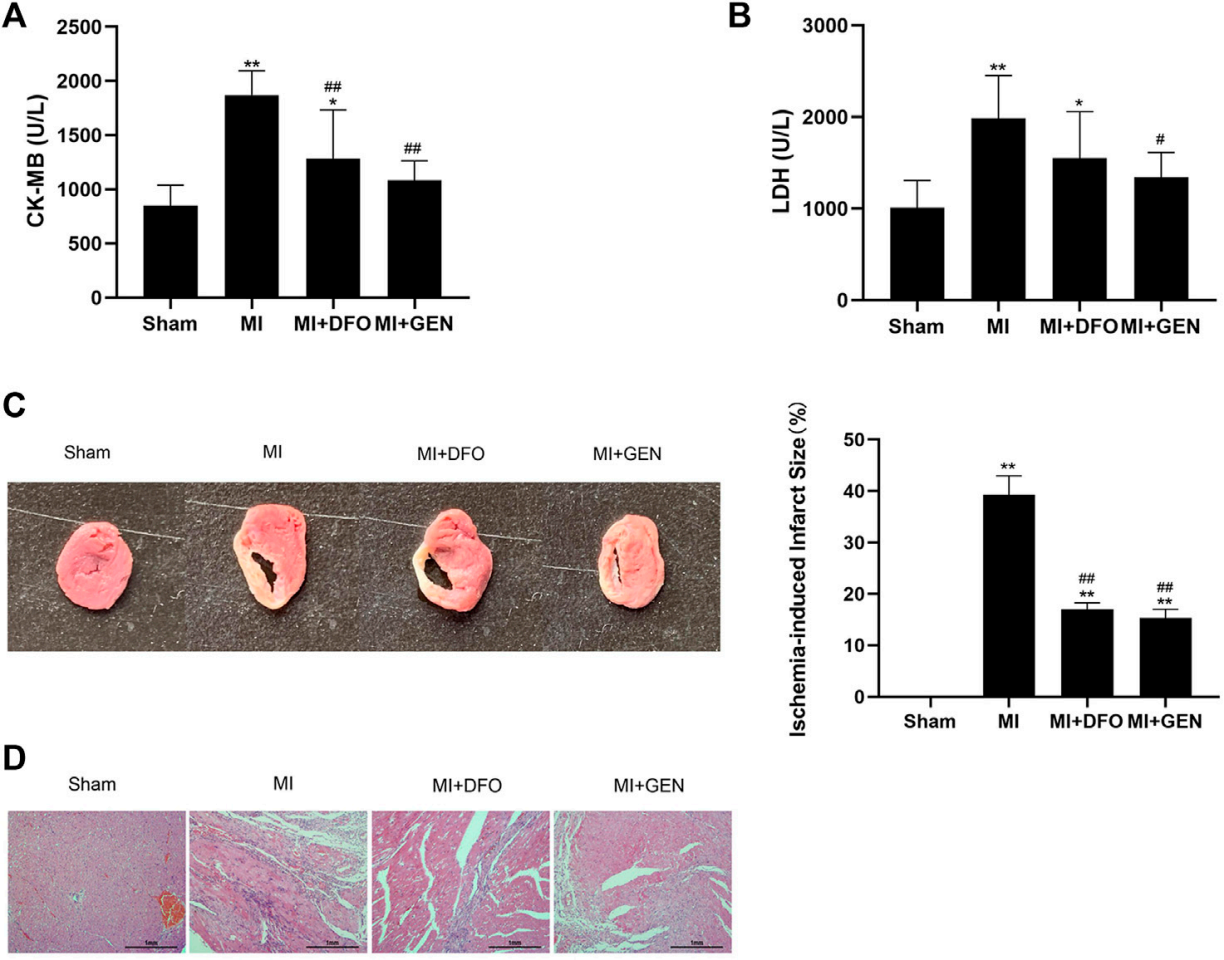
GEN improves cardiac pathological damages in rats. **(A,B)** Serum CK-MB and LDH levels in rats. **(C)** TTC staining of heart tissues to evaluate infarct size in rats of each group. **(D)** H&E staining of rat heart tissues in each group. Data were expressed as mean ± SD (*n* = 8). *p < 0.05, **p < 0.01 vs. Sham group; ^#^ *p* < 0.05, ^##^ *p* < 0.01 vs. MI group. Scale bar = 1 mm.

We measured several indicators of myocardial ischemic injury in rats. The levels of the myocardial enzymes CK-MB and LDH significantly increased in MI rats, which were reversed either by GEN or DFO (Figures 5A,B). In addition, GEN or DFO treatment narrowed down the ischemic infarction area in MI rats (Figure 5C). H&E staining revealed serious pathological changes in MI rats, manifesting as disordered myocardial cells in a vague and swollen shape, and pronounced infiltration of inflammatory factors (Figure 5D). In contrast, in Sham rats, myocardial muscle fibers were regularly arranged with a normal structure. These pathological changes were alleviated in MI rats treated with GEN or DFO, indicating their therapeutic effects on myocardial injury (Figure 5D).

## Discussion

To our knowledge, this is the first time to demonstrate the important pathogenesis of ferroptosis in aggravating myocardial injury, and the protective effects of GEN on myocardial ferroptosis. Here, our study indicated that ferroptosis induced by iron overload resulted in myocardial oxidative injury in cardiomyocytes and GEN showed cardioprotection by preventing ferroptosis and lipid peroxidation. Based on the results, we explored the effects and machanisms of GEN on ferroptosis caused by myocardial oxidative stress injury *in vitro*. The iron accumulation, reduced antioxidant capacity, massive ROS production, lipid peroxidation and mitochondrial dysfunction were the characteristics of ferroptosis, which were measured in H_2_O_2_-induced cardiomyocytes and rescued by GEN. Mechanistically, we found that activation of the Grsf1/GPx4 axis by GEN treatment inhibited myocardial ferroptosis and oxidative stress, providing a cardioprotective effect. Consistently, GEN treatment significantly alleviated cardiac injury by preventing ferroptosis and oxidative stress in MI rats *in vivo.* Our results suggested that GEN could be a promising drug candidate for ischemic heart disease.

Ischemic heart disease mainly characterized by myocardial ischemia and hypoxia is the leading cause of death worldwide. Generated ROS in the ischemic area put the impaired myocardium in an oxidative stress microenvironment. Thus, myocardial ischemia is essentially concerned with oxidative damage, which is also closely associated with the occurence of ferroptosis (Yan et al., 2020). Ferroptosis, driven by iron-dependent lipid ROS, that reportedly is an important form of cell death in myocardial injury (Dixon et al., 2012; Huang et al., 2021). A recent study showed that ferroptosis induced by abnormal iron metabolism due to MI is an important factor for left ventricular remodeling (Baba et al., 2018). Although increased studies have linked ferroptosis with myocardial injury, the exact effect and mechanism of ferroptosis on the ischemic myocardium remain unclear. Herein, our study proposed that ferroptosis aggravated myocardial oxidative injury and targeting ferroptosis to prevent cardiac cell death was an effective cardioprotective strategy.

Iron accumulation triggers ferroptosis, which is associated with the imbalance of iron homeostasis. Although it is well known that myocardial ischemia can induce changes in iron metabolism leading to iron overload, less attention has been paid to the role of iron in heart disease. Iron is an essential trace element for human metabolism, which widely participates in important physiological functions like electron transport, cell respiration, and DNA synthesis (Abbaspour et al., 2014). However, excess free iron ions possessing redox activity can induce myocardial oxidative stress and attack cellular membranes, proteins, and nucleic acids (Pantopoulos et al., 2012). An abnormal increase in myocardial iron poses a negative influence on cardiomyocytes, which was verified in our study. We first found that FAC-induced iron overload resulted in ferroptosis and severely impaired the cardiomyocytes, providing evidence of myocardial injury caused by iron overload. Further, we observed that H_2_O_2_-induced cardiomyocytes released more iron ions, which accelerated cardiomyocyte injury. Abundant Prussian blue-positive cells also reflected the strong iron deposition in myocardial tissues of MI rats. These findings emphasized the harmful effects of iron accumulation on the ischemic myocardium and the importance of maintaining iron homeostasis.

Iron importer Tfr1 and iron storage protein Fth1 play important roles in regulating iron homeostasis in cells and tissues. Tfr1 regulates the absorption of iron by importing extracellular iron into cells, providing the cellular iron pool required for the onset of ferroptosis (Feng H. et al., 2020). Fth1 is considered as an iron scavenger, which prevents cell damage by reducing iron-mediated ROS production *via* catalyzing the oxidation of Fe^2+^ to Fe^3+^ for storage in ferritin nanocages (Fang et al., 2020). In our study, Tfr1 expression was significantly upregulated, whereas Fth1 was downregulated in the myocardial oxidative stress models, implying that iron homeostasis was disturbed in myocardial injury.

Accumulated lipid ROS are executors of ferroptosis. An imbalance between myocardial oxygen consumption and myocardial oxygen delivery results in myocardial ischemia and ventricular dysfunction (Pagliaro et al., 2020). Impaired myocardium experiences oxidative challenge, which promotes the production and release of ROS (Kevin et al., 2005). Ferroptosis is closely associated with ROS production, which generates soluble and lipid ROS through iron-dependent enzymatic reactions (Jiang et al., 2020). Measuring lipid peroxides and oxidative stress products are necessary for determining whether ferroptosis occurs in myocardial injury. In the present study, excessive ROS and increased MDA and SA levels were observed in H_2_O_2_-induced primary cardiomyocytes and H9c2 cells. In addition, the decreased SOD activity indicated the reduced antioxidant capacity of cardiomyocytes. Furthermore, decreased mitochondrial ΔΨm in H_2_O_2_-induced cells suggested mitochondrial dysfunction that was unique to ferroptosis-induced morphological change. Consistently, increased MDA level was also observed in MI rats. Moreover, Ptgs2 is a key enzyme in prostaglandin biosynthesis, and also acts as a ferroptosis marker (Yang et al., 2014). It increases the activity of peroxidase and ROS level thereby regulating the oxidative state of the body. In our present study, the significant upregulation of Ptgs2 was observed both *in vitro* and *in vivo* models. Taken together, lipid ROS accelerated the process of myocardial injury, and inhibiting ferroptosis by suppressing lipid ROS may be a viable treatment approach.

GPx4 is a key enzyme responsible for suppressing lipid peroxidation. It requires GSH to reduce peroxides to their corresponding alcohols (Maiorino et al., 2018). As the main intracellular antioxidant, GSH synthesis disorder will lead to the loss of GPx4 activity and lipid ROS accumulation, which provides conditions for oxidative stress and ferroptosis (Lu et al., 2003; Maiorino et al., 2018). In H9c2 cells, inhibition of GPx4 can cause lipid peroxidation, boosting myocardial ferroptosis (Park et al., 2019). In this study, we observed a significant decrease in GPx4 expression in H_2_O_2_-induced cardiomyocytes and the heart tissues of MI rats. In addition, GSH levels decreased in MI model rats. These results demonstrated that downregulation of GPx4 expression reduced the ability to resist lipid peroxidation and enhanced the sensitivity to ferroptosis, causing more severe myocardial oxidative injury. Of note, GPx4 is also distributed in various organelles such as mitochondrion. Additional studies are required to elucidate the effect of mitochondrial GPx4 on ferroptosis since mitochondrion is the main site of iron metabolism and ROS generation.

Grsf1 is a mitochondrial RNA-binding protein involved in RNA splicing, stability maintenance, and translation initiation (Ufer, 2012). As a translational regulator of GPx4, Grsf1 modulates the cellular redox states (Ufer et al., 2008; Yin et al., 2019). Given the important role of Grsf1 in suppressing ROS production, we considered it essential to investigate the potential effect of Grsf1 on ferroptosis. We found that Grsf1 was downregulated in H_2_O_2_-induced cells and MI rats. To explore the role of Grsf1 in regulating ferroptosis *via* GPx4, ferroptosis-related changes were detected in H_2_O_2_-induced cells with Grsf1 knockdown. It is shown that knockdown of Grsf1 increased the sensitivity to ferroptosis in H_2_O_2_-induced myocardial cells because of the release of free iron and increased MDA level. Moreover, Ptgs2 and Tfr1 were significantly upregulated, whereas Fth1 was downregulated after knockdown of Grsf1. Importantly, GPx4 expression was also reduced, indicating a reduction in antioxidant capacity. Thus, knockdown of Grsf1 in H_2_O_2_-induced cardiomyocytes accelerated the process of myocardial ferroptosis by weakening the response to lipid peroxidation and oxidative stress, and promoting iron deposition, which further aggravated the oxidative damage of the myocardium.

GEN is a natural product extracted from the traditional Chinese herb *Gardenia jasminoides J. Ellis*. Although GEN is widely used in the treatment of ischemic heart disease owing to its protective properties of anti-inflammatory, antihyperlipidemic, antioxidative stress, and antithrombotic (Zhang et al., 2012; Li et al., 2019), the effects of GEN on myocardial ferroptosis have not been reported yet. To date, the prevention of ferroptosis by traditional Chinese medicine has been rarely reported. The present study demonstrated that GEN intervention significantly attenuated FAC- and H_2_O_2_-induced ferroptosis injury in primary cardiomyocytes and H9c2 cells. Moreover, using Fer-1 or DFO as a positive control, GEN administration significantly reversed the expression levels of ferroptosis-related proteins in cardiomyocytes, whose effects were strengthened when combined with Fer-1 or DFO. GEN or DFO treatment mitigated iron accumulation and oxidative stress injury and improved myocardial pathological damage in MI rats, thereby delaying the development of MI. More importantly, GEN activated the expression of Grsf1 both *in vitro* and *in vivo*. Considering that Grsf1 transcriptionally regulates GPx4, we speculated that GEN may improve the anti-oxidative stress capacity of damaged myocardium by regulating the Grsf1/GPx4 signaling pathway. Surprisingly, the cardioprotective effect of GEN was weakened after knockdown of Grsf1. Our study provided some objective evidence that GEN inhibited oxidative stress and ferroptosis by upregulating the Grsf1/GPx4 axis, thus protecting the injured myocardium. Moreover, the specific mechanism underlying the effects of Grsf1 on MI-induced ferroptosis *in vivo* should be explored in the future.

## Conclusion

**FIGURE 6:**
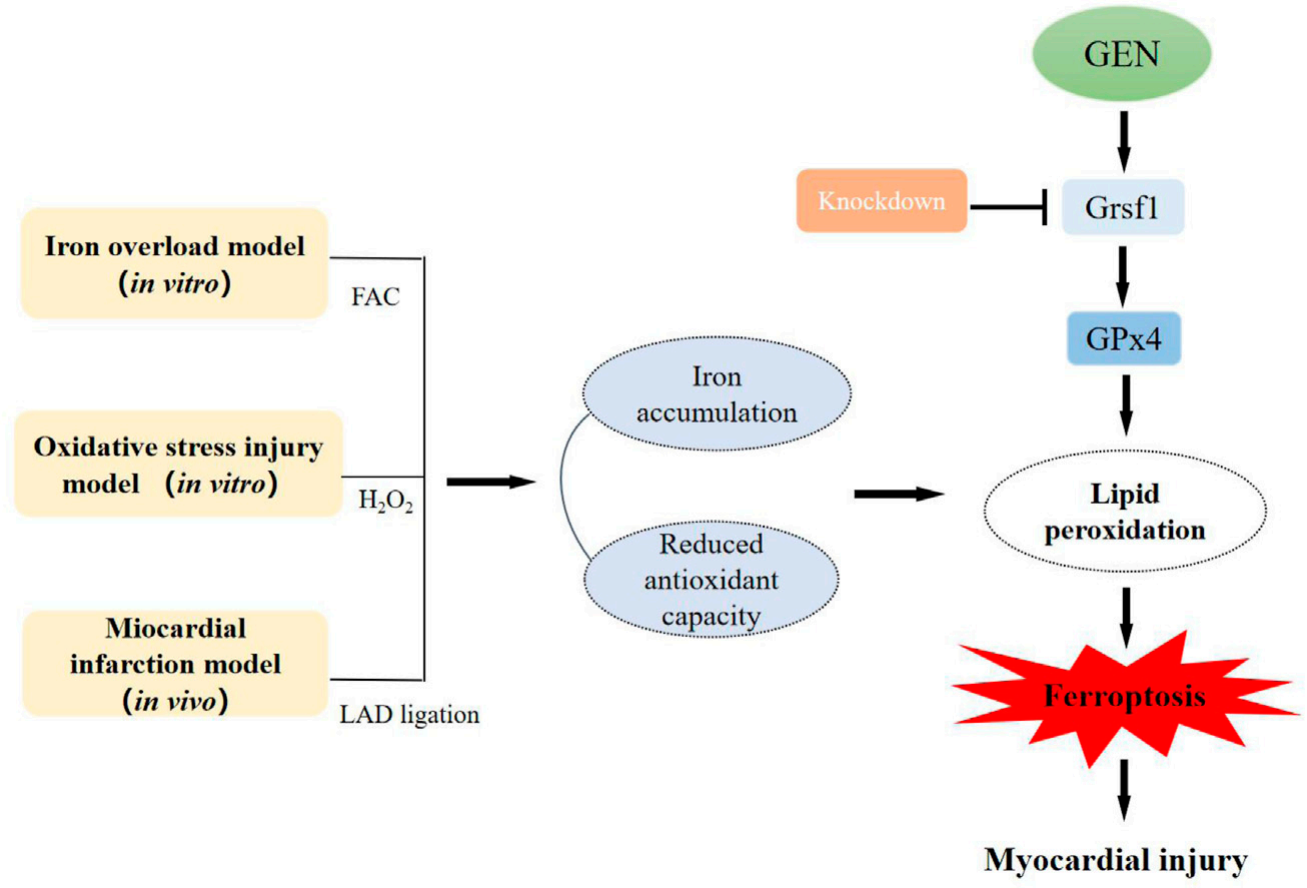
The pathogenesis of myocardial ferroptosis and the protective mechanism of GEN against myocardial injury *in vitro* and *in vivo*.

In conclusion, we for the first time demonstrated that ferroptosis plays a crucial role in the development of ischemic myocardium in an oxidative stress microenvironment, and GEN exerts a cardioprotective role by inhibiting oxidative stress and ferroptosis *via* activating the Grsf1/GPx4 axis (Figure 6). Thus, GEN is a promising therapeutic drug for ischemic heart disease and is worthy of further study.

## Data Availability

The original contributions presented in the study are included in the article/Supplementary Material, further inquiries can be directed to the corresponding authors.
