# Supplementary material for: Geniposide Possesses the Protective Effect on Myocardial Injury by Inhibiting Oxidative Stress and Ferroptosis via Activation of the Grsf1/GPx4 Axis
Source: Front Pharmacol. 2022 May 5;13:879870. doi: 10.3389/fphar.2022.879870 (PMC9117627; doi:10.3389/fphar.2022.879870)
Supplement: Supplementary file 1 [file DataSheet1.PDF]

Supplementary Material

Supplementary Figure S1 | Effects of GEN on characteristics of ferroptosis in FAC-induced cardiomyocytes treated with GEN.

Supplementary Figure S2 | Effects of GEN on characteristics of ferroptosis in H_2O_2-induced cardiomyocytes.

Supplementary Figure S3 | Effects of GEN and Fer-1 on ferroptosis-related proteins expression in H_2O_2-induced cardiomyocytes.

Supplementary Figure S4 | Effects of GEN and DFO on ferroptosis-related proteins expression in H_2O_2-induced cardiomyocytes.

Supplementary Figure S5 | Quantitative analysis of Grsf1 expression in H_2O_2-induced cardiomyocytes with GEN treatment, and Grsf1 and GPx4 expression in H_2O_2-treated H9c2 cells after knockdown of Grsf1.

Supplementary Figure S6 | Analysis of Fe^{2+} fluorescence intensity and quantification of ferroptosis-related proteins Ptgs2, Fth1, and Tfr1 in H_2O_2-induced cardiomyocytes with GEN treatment after knockdown of Grsf1.

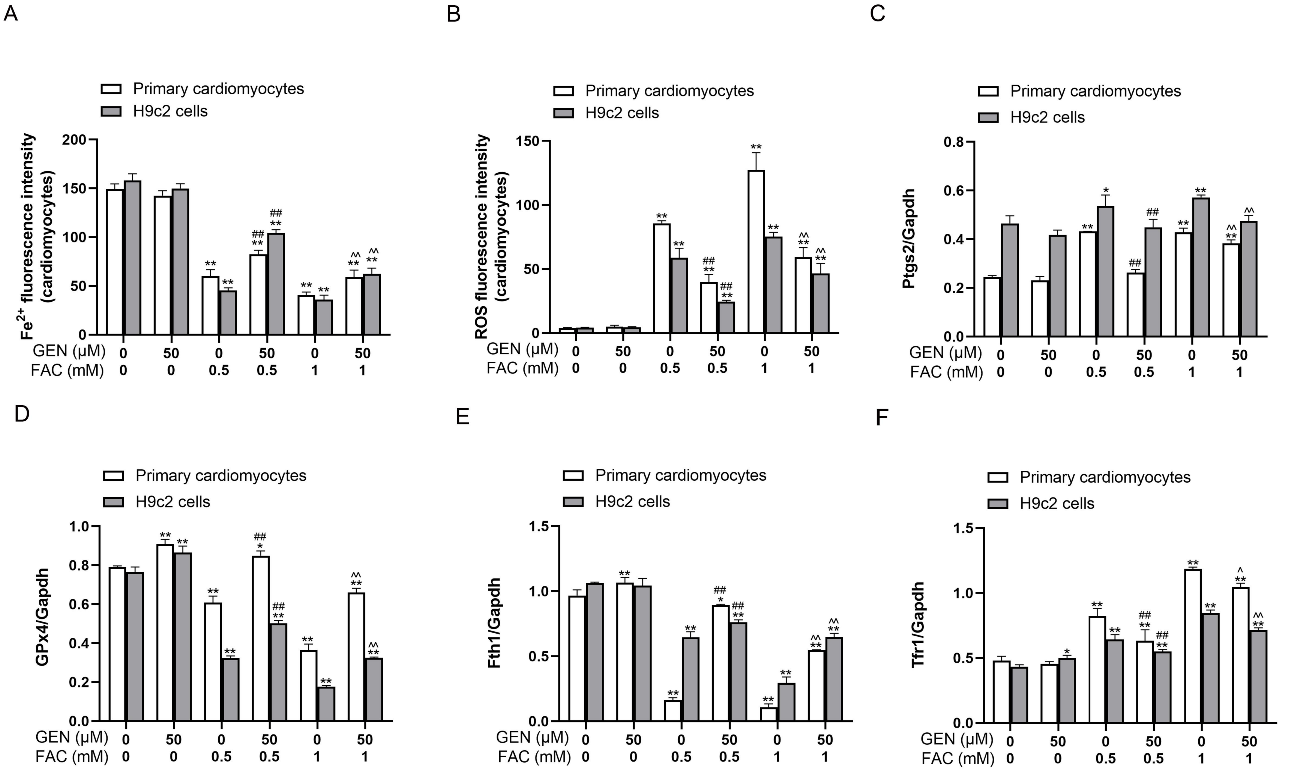

FIGURE S1 | Effects of GEN on characteristics of ferroptosis in FAC-induced cardiomyocytes treated with GEN. Supplemental to FIGURE 1. **(A,B)** Analysis of Fe^{2+} and ROS fluorescence intensity in primary cardiomyocytes and H9c2 cells with GEN treatment using imageJ. **(C,D,E,F)** Quantitative analysis of western blot results of Ptgs2, GPx4, Fth1, and Tfr1 in primary cardiomyocytes and H9c2 cells with GEN treatment. Data are shown as mean±SD (n = 5). **p*<0.05, ***p*<0.01 vs. Control group; #*p*<0.05, ##*p*<0.01 vs. 0.5 mM FAC group; ^*p*<0.05, ^^*p*<0.01 vs. 1 mM FAC group.

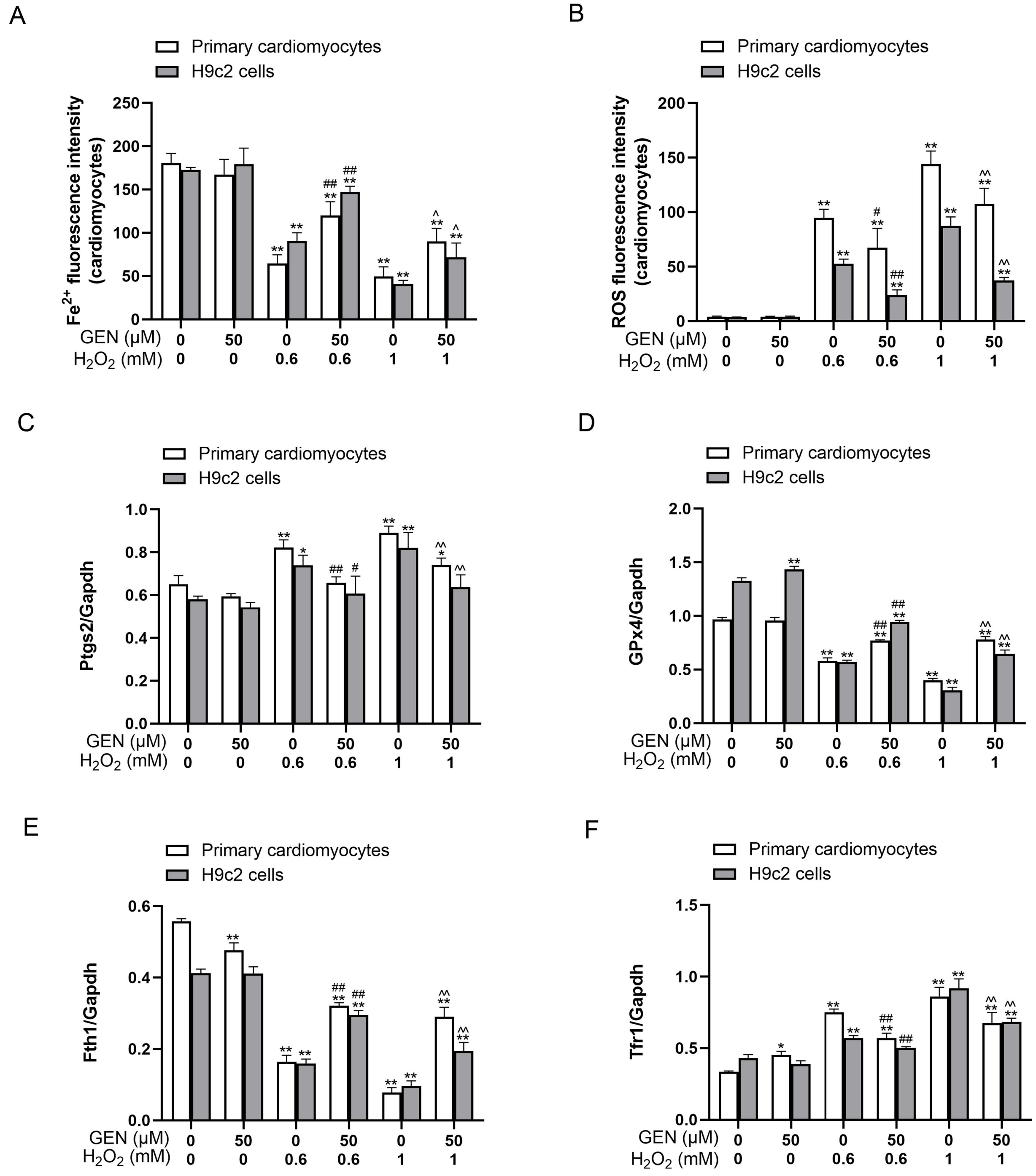

FIGURE S2 | Effects of GEN on characteristics of ferroptosis in H_2O_2-induced cardiomyocytes. Supplemental to FIGURE 2. **(A, B)** Analysis of Fe^{2+} and ROS fluorescence intensity in primary cardiomyocytes and H9c2 cells with GEN treatment using imageJ. **(C,D,E,F)** Quantitative analysis of western blot results of Ptgs2, GPx4, Fth1, and Tfr1 in primary cardiomyocytes and H9c2 cells with GEN treatment. Data are shown as mean±SD (n = 5). **p*<0.05, ***p*<0.01 vs. Control group; #*p*<0.05, ##*p*<0.01 vs. 0.6 mM H_2O_2 group; ^*p*<0.05, ^^*p*<0.01 vs. 1 mM H_2O_2 group.

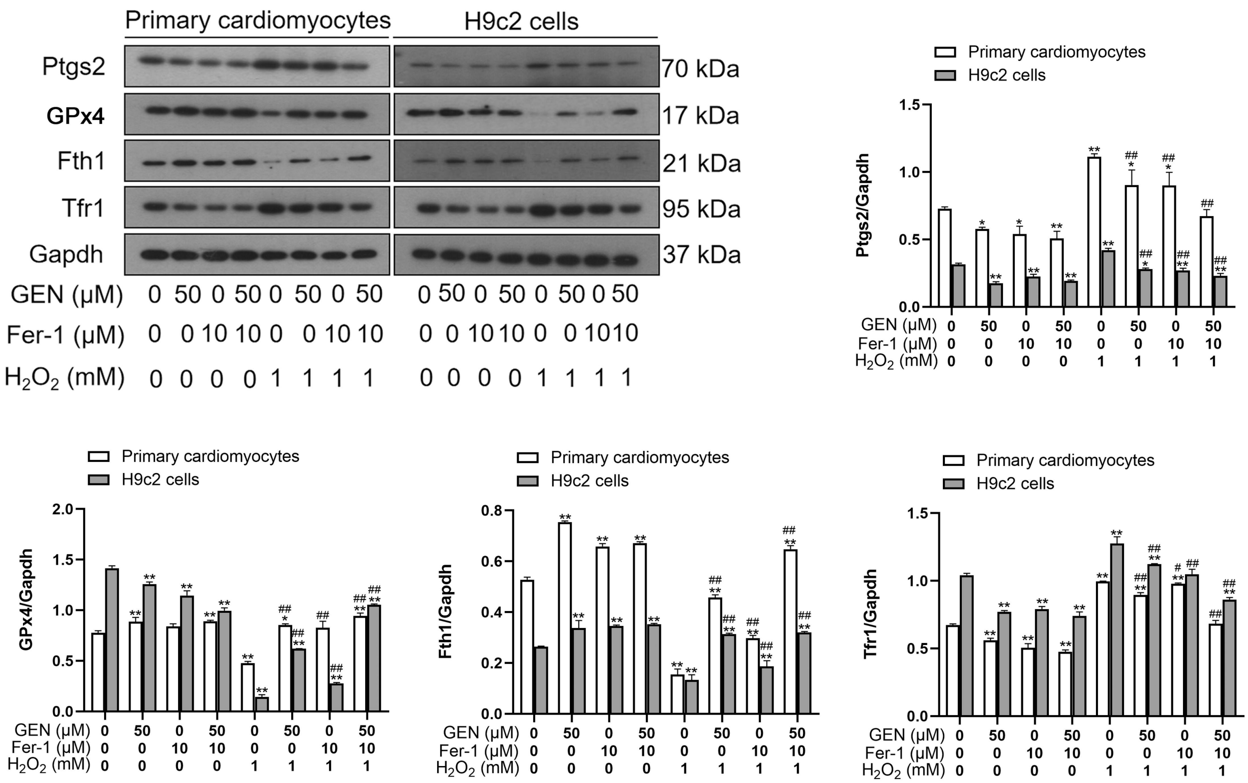

FIGURE S3 | Effects of GEN and Fer-1 on ferroptosis-related proteins expression in H_2O_2-induced cardiomyocytes. Supplemental to FIGURE 2. Quantification of Ptgs2, GPx4, Fth1, and Tfr1 in primary cardiomyocytes and H9c2 cells treated or cotreated with GEN and Fer-1 after H_2O_2 induction. Data are shown as mean±SD (n = 5). **p*<0.05, ***p*<0.01 vs. Control group; #*p*<0.05, ##*p*<0.01 vs. 1 mm H_2O_2 group.

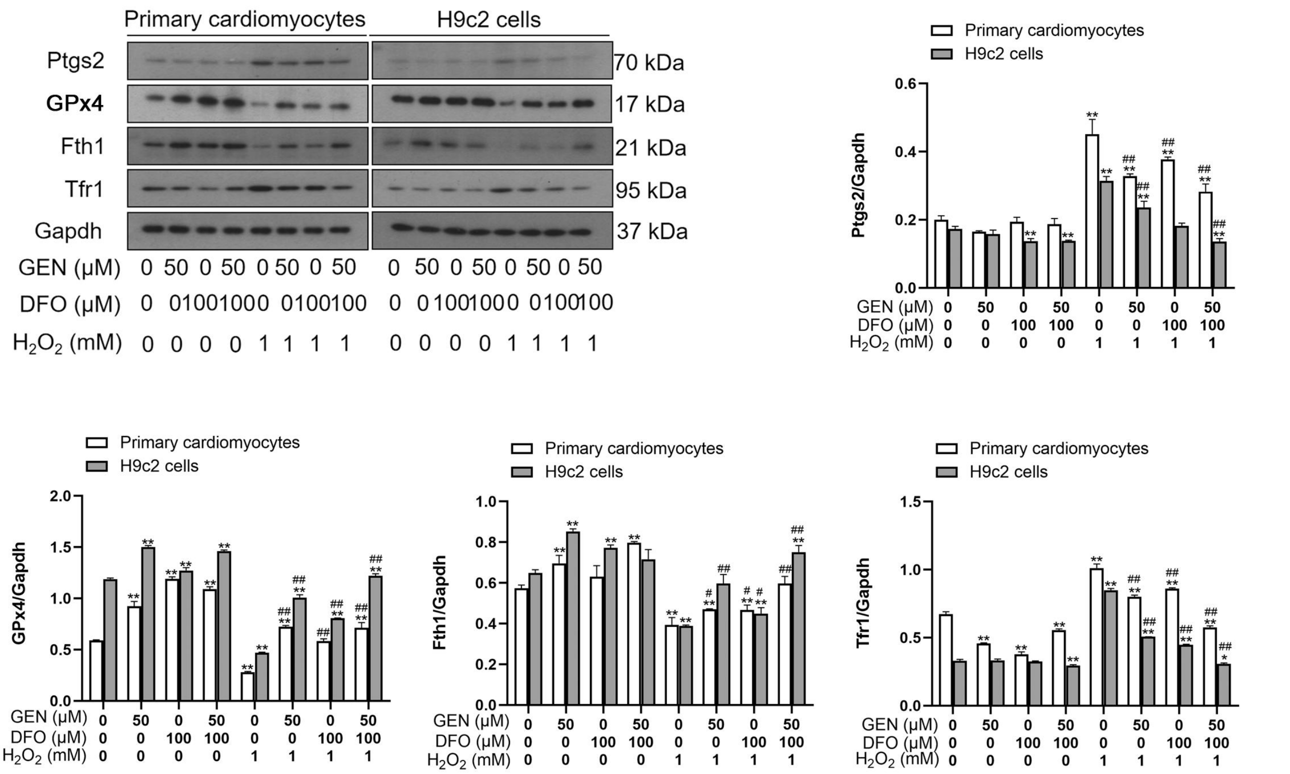

FIGURE S4 | Effects of GEN and DFO on ferroptosis related-proteins expression in H_2O_2-induced cardiomyocytes. Supplemental to FIGURE 2. Quantification of Ptgs2, GPx4, Fth1, and Tfr1 in primary cardiomyocytes and H9c2 cells treated or cotreated with GEN and DFO after H_2O_2 induction. Data are shown as mean±SD (n = 5). $^{**}p<0.01$ vs. Control group; $\#p<0.05$, $\#\#p<0.01$ vs. 1 mm H_2O_2 group.

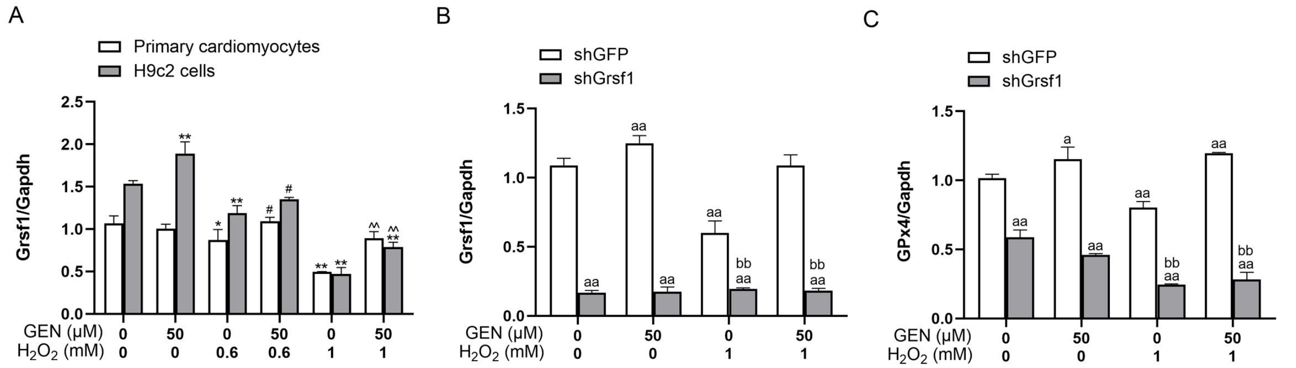

FIGURE S5 | Quantitative analysis of Grsf1 expression in H_2O_2-induced cardiomyocytes with GEN treatment, and Grsf1 and GPx4 expression in H_2O_2-treated H9c2 cells after knockdown of Grsf1. Supplemental to FIGURE 3. **(A,B,C)** Quantification of Grsf1 expression before knockdown of Grsf1, and expression of Grsf1 and GPx4 after knockdown of Grsf1 in H_2O_2-induced cells treated with GEN. Data are shown as mean±SD (n = 5). $^{*}p<0.05$, $^{**}p<0.01$ vs. Control group; $\#p<0.05$, vs. 0.6 mM

H_2O_2 group; ^^p<0.01 vs. 1 mM H_2O_2 group; [a]p<0.05, [aa]p<0.01 vs. shGFP control group; [bb]p<0.01, shGrsf1 group vs. shGFP group.

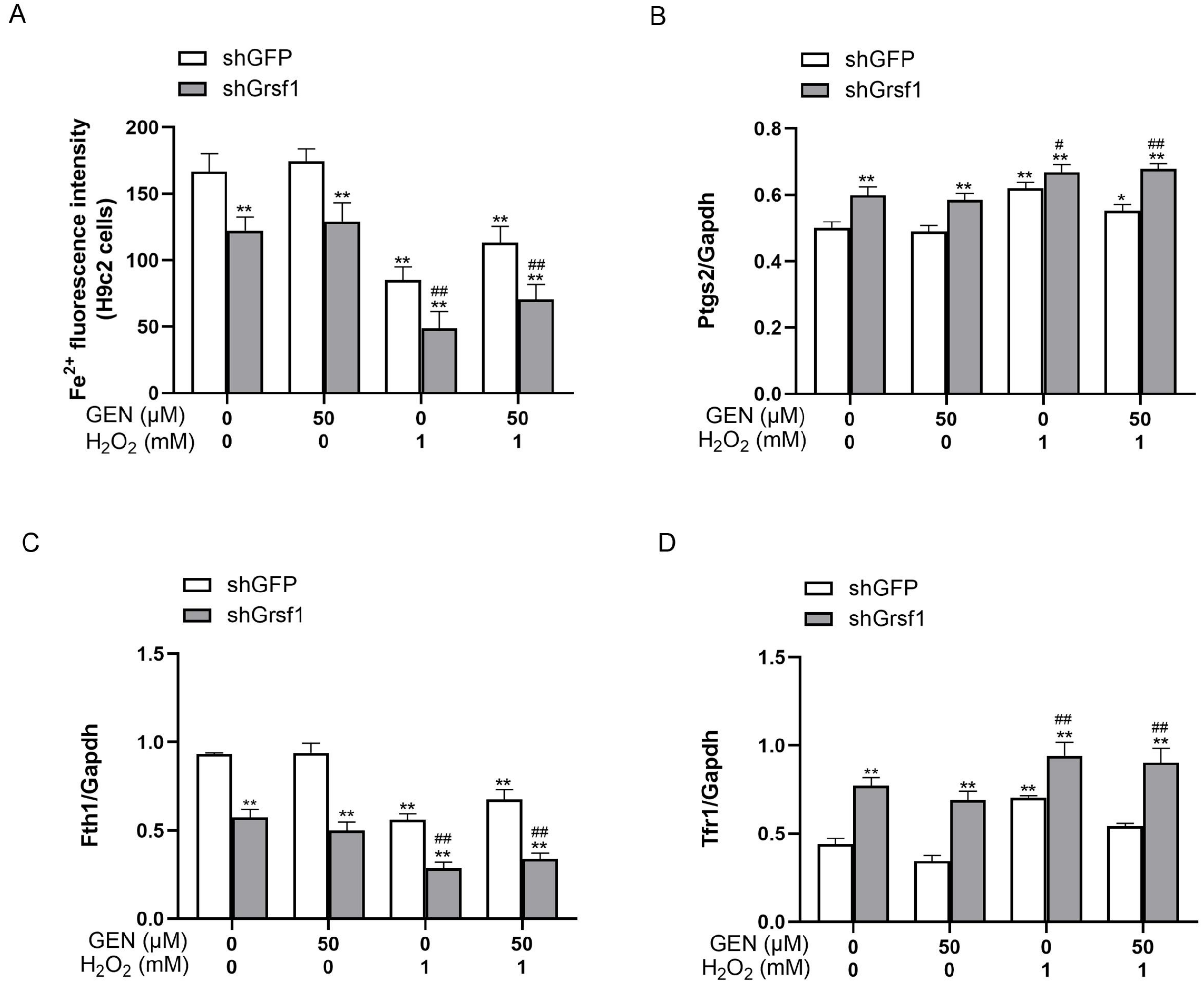

FIGURE S6 | Analysis of Fe^{2+} fluorescence intensity and quantification of ferroptosis-related proteins Ptgs2, Fth1, and Tfr1 in H_2O_2-induced cardiomyocytes with GEN treatment after knockdown of Grsf1. Supplemental to FIGURE 3. **(A)** Analysis of Fe^{2+} level in H_2O_2-induced cells treated with GEN. **(B,C,D)** Quantification of immunoblotting results of Ptgs2, Fth1, and Tfr1 in H_2O_2-induced cells treated with GEN. Data are shown as mean±SD (n = 5). *p<0.05, **p<0.01 vs. shGFP control group; #p<0.05, ##p<0.01, shGrsf1 group vs. shGFP group.
